# Disparities in Who Receives Weight-Loss Advice From a Health Care Provider: Does Income Make a Difference?

**DOI:** 10.5888/pcd13.160183

**Published:** 2016-10-06

**Authors:** Cori Lorts, Punam Ohri-Vachaspati

**Affiliations:** Author Affiliation: Cori Lorts, School of Nutrition and Health Promotion, Arizona State University, Phoenix, Arizona.

## Abstract

**Introduction:**

The US Preventive Services Task Force recommends that all patients be screened for obesity and, if needed, be provided weight-loss advice. However, the prevalence of such advice is low and varies by patient demographics. This study aimed to describe the determinants of receiving weight-loss advice among a sample with a high proportion of low-income, racial/ethnic minority individuals.

**Methods:**

Data were collected from a telephone survey of 1,708 households in 2009 and 2010 in 5 cities in New Jersey. Analyses were limited to 1,109 overweight or obese adults. Multivariate logistic regression determined the association of participants’ characteristics with receiving weight-loss advice from their health care provider. Two models were used to determine differences by income and insurance status.

**Results:**

Of all overweight or obese respondents, 35% reported receiving advice to lose weight. Receiving advice was significantly associated with income in multivariate analysis. Compared with those with an income at or below 100% of the federal poverty level (FPL), those within 200% to 399% of the FPL had 1.60 higher odds of receiving advice (*P* = .02), and those with an income of 400% or more of the FPL had 1.73 higher odds of receiving advice (*P* = .03). The strength of the association did not change after adjusting for health insurance.

**Conclusion:**

Income is a significant predictor of whether or not overweight or obese adults receive weight-loss advice after adjustment for demographic variables, health status, and insurance status. Further work is needed to examine why disparities exist in who receives weight-loss advice. Health care providers should provide weight-loss advice to all patients, regardless of income.

**MEDSCAPE CME**

Medscape, LLC is pleased to provide online continuing medical education (CME) for this journal article, allowing clinicians the opportunity to earn CME credit.

This activity has been planned and implemented through the joint providership of Medscape, LLC and *Preventing Chronic Disease*. Medscape, LLC is accredited by the American Nurses Credentialing Center (ANCC), the Accreditation Council for Pharmacy Education (ACPE), and the Accreditation Council for Continuing Medical Education (ACCME), to provide continuing education for the healthcare team.

Medscape, LLC designates this Journal-based CME activity for a maximum of 1.00 ***AMA PRA Category 1 Credit(s)™***. Physicians should claim only the credit commensurate with the extent of their participation in the activity.

All other clinicians completing this activity will be issued a certificate of participation. To participate in this journal CME activity: (1) review the learning objectives and author disclosures; (2) study the education content; (3) take the post-test with a 75% minimum passing score and complete the evaluation at http://www.medscape.org/journal/pcd; (4) view/print certificate.

Release date: October 6, 2016; Expiration date: October 6, 2017

**Learning Objectives**

Upon completion of this activity, participants will be able to:

1. Distinguish the rate of advice from healthcare providers to lose weight in the current study
2. Evaluate demographic variables associated with higher rates of advice to lose weight in the current study
3. Analyze the effect of race and ethnicity on the rate of advice to lose weight in the current study
4. Determine the effects of patient income and health insurance status on the rate of advice to lose weight in the current study

**EDITOR**

Ellen Taratus, MS, ELS

Editor, Preventing Chronic Disease

Disclosure: Ellen Taratus has disclosed no relevant financial relationships.

 **CME AUTHOR**

Charles P. Vega, MD

Health Sciences Clinical Professor of Family Medicine, University of California, Irvine, California

Disclosure: Charles P. Vega, MD, has disclosed the following relevant financial relationships:

Served as an advisor or consultant for: Allergan, Inc.; McNeil Consumer Healthcare

Served as a speaker or a member of a speakers bureau for: Shire

 **AUTHORS**

Cori Lorts, MPH, RD

School of Nutrition and Health Promotion, Arizona State University, Phoenix, Arizona

Disclosure: Cori Lorts, MPH, RD, has disclosed no relevant financial relationships.

 Punam Ohri-Vachaspati, PhD, RD

School of Nutrition and Health Promotion, Arizona State University, Phoenix, Arizona

Disclosure: Punam Ohri-Vachaspati, PhD, RD, has disclosed no relevant financial relationships.

## Introduction

Despite the US Preventive Services Task Force’s recommendation that all patients be screened for obesity and provided weight-loss advice if needed (1), the prevalence of such advice is low and ranges from 34% to 65% among overweight and obese populations (2–4). The low rate of weight-loss advice is problematic; patients who receive weight-loss advice are more likely to decrease their caloric intake, eat less fat (5,6), and lose more weight (2,7) than patients who do not receive weight-loss advice.

High-risk patients, such as those who are extremely obese or have comorbidities, are most likely to receive weight-loss advice (3,8,9). However, demographic factors also appear to determine who receives advice. Patients who have high levels of education are more likely to receive advice than those who have low levels of education (3,10), and those who are middle-aged are more likely to receive advice than younger or older patients (10,11). Study results are unclear about whether health insurance plays a role in determining who receives weight-loss advice; some studies found that insurance is not associated with receiving weight-loss advice (11,12), whereas one study found that patients who had private insurance were more likely than uninsured patients to receive weight-loss advice (13). Income also appears to play a role; those who have high incomes are more likely to receive weight-loss advice than those who have low incomes (6, 14).

Most research that examined determinants of receiving weight-loss advice studied a majority-white population (8–11,13). Our study is the first to explore the determinants of receiving weight-loss advice in a sample of overweight or obese individuals from communities with a high proportion of low-income, racial/ethnic minority populations in a multivariate analysis of race/ethnicity, age, sex, health status, income, health insurance status, and education.

## Methods

The data used in this study were collected from a random-digit–dial telephone survey of 1,708 households in 2009 and 2010 in 5 cities in New Jersey: Camden, Newark, New Brunswick, Trenton, and Vineland. The survey respondent was the adult who made most of the food purchasing decisions for the household. Each interview took on average 36 minutes to complete, and participants were given $10 for their participation. Interviews were conducted in English and Spanish, and only households with at least 1 child aged 3 to 18 were selected. The participation rate was 49%, similar to the response rate (50%) for the New Jersey Behavioral Risk Factor Surveillance System Survey for 2010 (15). All study procedures were approved by Rutgers University and Arizona State University institutional review boards.

The survey included items on health status, height and weight, food environment, physical activity environment, child health behaviors, adult health behaviors, health insurance, employment and earnings, and demographics. For this study, we used data on health status, height and weight, health insurance, employment and earnings, and demographics, all of which were self-reported.

Our analysis was limited to 1,109 overweight or obese adults who had complete data on all variables of interest (65% of the study population [1,109 of 1,708]). Overweight was categorized as a body mass index (BMI, kg/m^2^) of 25.0–29.9, obesity was categorized as a BMI of 30.0–39.9, and extreme obesity was categorized as a BMI of 40.0 or greater (16). BMI was calculated from self-reported height and weight. Self-reported BMI is highly correlated with objectively measured BMI (17).

The outcome variable was whether or not the respondent reported receiving advice to lose weight from their health care provider within the previous 12 months, determined by the survey question, “In the past 12 months, has a doctor, nurse, or other health professional given you advice about your weight?” Responses categories included “yes, lose weight,” “yes, gain weight,” “yes, maintain weight,” “no advice given about weight,” “don’t know/not sure,” or refusal to answer. Those who responded “yes, lose weight” were coded as 1 for health care provider’s advice to lose weight while those who received no advice were coded as 0. Those who responded with “yes, gain weight” or “yes, maintain weight” were excluded from the sample because of conflicting responses with a self-reported BMI indicating overweight or obesity (n = 32).

Information on age, sex, race/ethnicity, income, health insurance, education, BMI, general health status, and previous diagnosis of diabetes or asthma were collected from each respondent. Income was categorized according to a ratio of federal poverty level (FPL) to account for differences in income based on household size. General health status was determined by the survey question “Would you say your health is . . . ,” and possible responses were excellent, very good, good, fair, or poor. Respondents could also refuse to answer or say they were unsure; these data were treated as missing and data for these respondents were excluded from the analysis. Responses of excellent and very good were combined into a single group, excellent/very good, as were responses of fair and poor into fair/poor. Previous diabetes and asthma diagnoses were determined by the question “Has a doctor or other health professional ever said that you had (diabetes/asthma)?” Possible responses were yes, no, or “don’t know” or refusal to answer. Health insurance status was determined by asking the respondent, “Do you have some form of health insurance or health care coverage, or not?” If the participant responded yes, they had insurance, they were asked, “Are you mainly covered by Medicare, Medicaid, NJ FamilyCare, insurance through a current or former job or other private insurance, or do you have coverage from some other source?” Responses were categorized as Medicare, Medicaid, or “other,” which included private insurance, employer-provided insurance, or other insurance. Given an expected collinearity between income and government insurance (Medicaid), preliminary analysis was conducted with these 3 groups. The odds ratios were similar for all types of insurances compared with no insurance; thus for parsimony, insurance status was dichotomized as uninsured or insured.

Descriptive analyses were conducted on all variables to examine their distribution. Bivariate analyses were conducted using χ^2^ tests for categorical variables. Multivariate logistic regression analyses determined the association of participants’ characteristics with receiving a health care provider’s advice to lose weight. Independent variables included in the models were based on previous research and their significant bivariate associations with the dependent variable. Two multivariate models were used. Model 1 adjusted for respondent age, sex, race/ethnicity, BMI, income (as a poverty ratio), education, general health, diabetes diagnosis, and asthma diagnosis. To investigate whether health insurance status influenced any of the associations, Model 2 adjusted for insurance status along with other covariates in Model 1. All analyses were conducted in Stata version 13.1 (StataCorp LP) and were considered significant at *P* < .05.

## Results

The sample was mostly female, racial/ethnic minority, and low income (Table 1). Half (51%) of the sample was non-Hispanic black and 38% were Hispanic. One-third (33%) of households were at or below 100% of the FPL, 30% were between 100% and 199% of the FPL, 23% were between 200% and 399% of the FPL, and 14% were at or above 400% of the FPL. Fifteen percent of the sample had received a previous diabetes diagnosis, and 22% had received a previous asthma diagnosis. Less than one-fifth (17%) of participants had no insurance and 83% had some form of insurance. Of these 83%, 7% were covered by Medicare, 32% had Medicaid, and 44% had employer-provided insurance, private insurance, or some other form of insurance.

**Table 1:** Demographic and Health Characteristics for Overweight and Obese Respondents and by Whether They Received Advice to Lose Weight From a Health Care Provider in the Previous 12 Months^a^^,^^b^

Characteristic	All Respondents, No. (%^c^) (n = 1,109)	By Advice to Lose Weight		
		Did Not Receive Advice, No. (%^d^) (n = 720)	Received Advice, No. (%^d^) (n = 389)	*P* Value for Difference^e^
**Age, y^f^**				
18–34	329 (30)	240 (73)	89 (27)	.001
35–54	648 (58)	403 (62)	245 (38)	
≥55	132 (12)	77 (58)	55 (42)	
**Sex^f^**				
Male	218 (20)	160 (73)	58 (27)	.003
Female	891 (80)	560 (63)	331 (37)	
**Race/ethnicity^f^**				
Non-Hispanic white	128 (12)	93 (73)	35 (27)	.14
Non-Hispanic black	562 (51)	356 (63)	206 (37)	
Hispanic	419 (38)	271 (65)	148 (35)	
**Education^f^^, ^ g**				
<High school	179 (16)	125 (70)	54 (30)	.004
High school equivalent	438 (40)	287 (66)	151 (34)	
Some college	303 (28)	174 (57)	129 (43)	
College degree	178 (16)	128 (72)	50 (28)	
**Body mass index^h^**				
25.0–29.9 (overweight)	500 (45)	388 (78)	112 (22)	.65
30.0–39.9 (obesity)	507 (46)	294 (58)	213 (42)	
≥40.0 (extreme obesity)	102 (9)	38 (37)	64 (63)	
**Poverty ratio as a percentage of federal poverty level^i^**				
≤100	368 (33)	246 (67)	122 (33)	.65
100–199	331 (30)	213 (64)	118 (36)	
200–399	259 (23)	161 (62)	98 (38)	
≥400	151 (14)	100 (66)	51 (34)	
**Has health insurance^f^**				
No	190 (17)	149 (78)	41 (22)	<.001
Yes	919 (83)	571 (62)	348 (38)	
**General health^f^**				
Excellent/very good	441 (40)	323 (73)	118 (27)	<.001
Good	411 (37)	261 (64)	150 (36)	
Fair/poor	257 (23)	136 (53)	121 (47)	
**Diabetes diagnosis^f^**				
No	943 (85)	645 (68)	298 (32)	<.001
Yes	166 (15)	75 (45)	91 (55)	
**Asthma diagnosis^f^**				
No	870 (78)	588 (68)	282 (32)	<.001
Yes	239 (22)	132 (55)	107 (45)	

a Data collected from random-digit–dial telephone surveys in 5 cities in New Jersey in 2009 and 2010: Camden, Newark, New Brunswick, Trenton, and Vineland.

b All values are number (percentage).

c Percentages may not sum to 100% because of rounding.

d Percentages add to 100% across 2 columns (received advice and did not receive advice). Percentages may not sum to 100% because of rounding.

e χ^2^ analysis used to determine *P* values for differences between respondents who received advice to lose weight and those who did not.

f Self-reported.

g Eleven respondents did not answer the question on education.

h Body mass index calculated as kg/m^2^ according to self-reported height and weight.

i Poverty ratio calculated according to self-reported household income.

Of all overweight or obese respondents, 35% reported receiving a health care provider’s advice to lose weight. As BMI increased, so did the prevalence of receiving weight-loss advice; only 22% of those with a BMI of 25.0 to 29.9 received advice, 42% of those with a BMI of 30.0 to 39.9 received advice, and 63% of those with a BMI of 40.0 or greater received advice.

In Model 1 of the multivariate logistic regression analyses, receiving a health care provider’s advice was significantly associated with income (Table 2).Compared with respondents who had a household income at or below 100% of the FPL, respondents who had a household income within 200% to 399% of the FPL had 1.60 higher odds of receiving advice (*P* = .02), and respondents who had a household income of 400% of the FPL or greater had 1.73 higher odds of receiving advice (*P* = .03). Hispanics had almost twice the odds (odds ratio = 1.88, *P* = .01) of receiving health care provider advice to lose weight than non-Hispanic whites and the odds were similar for non-Hispanic whites and non-Hispanic blacks. Receiving a health care provider’s advice was also significantly associated with health status; compared with respondents who reported their health as very good or excellent, respondents who reported their health as good had 1.41 higher odds of receiving weight-loss advice (*P* = .03), and respondents who reported their health as fair or poor had a 1.94 higher odds of receiving weight-loss advice (*P* = .001). Respondents who had a diabetes diagnosis had significantly higher odds of receiving advice than respondents who did not have a diabetes diagnosis, and those who had an asthma diagnosis had significantly higher odds of receiving advice than those who did not have one. Obese respondents had higher odds of receiving weight-loss advice; compared with respondents who had a BMI of 25.0 to 29.9, respondents who had a BMI of 30.0 to 39.9 had 2.34 higher odds of receiving advice (*P* < .001), and respondents who had a BMI of 40.0 or greater had 5.21 higher odds of receiving advice (*P* < .001).

**Table 2:** Adjusted Odds Ratios for Receiving Advice to Lose Weight From a Health Care Provider, by Demographic and Health Characteristics for All Overweight or Obese Study Respondents^a^^,^^b^

Characteristic	Model 1 (Without Insurance)		Model 2 (With Insurance)	
	OR (95% CI)	*P* Value	OR (95% CI)	*P* Value
**Age, y^c^**				
18–34	1 [Reference]		1 [Reference]	
35–54	1.47 (1.07–2.03)	.02	1.43 (1.04–1.98)	.03
≥55	1.38 (0.85–2.24)	.19	1.31 (0.8–2.13)	.28
**Sex^c^**				
Male	1 [Reference]		1 [Reference]	
Female	1.56 (1.08–2.25)	.02	1.45 (0.998–2.1)	.05
**Race/ethnicity^c^**				
Non-Hispanic white	1 [Reference]		1 [Reference]	
Non-Hispanic black	1.37 (0.86–2.19)	.19	1.36 (0.85–2.18)	.20
Hispanic	1.88 (1.15–3.07)	.01	2.04 (1.24–3.34)	.005
**Education^c^**				
<High school	1 [Reference]		1 [Reference]	
High school equivalent	1.44 (0.95–2.19)	.09	1.38 (0.91–2.12)	.13
Some college	1.98 (1.24–3.13)	.004	1.85 (1.16–2.95)	.01
College degree	1.11 (0.65–1.92)	.70	1.01 (0.58–1.75)	.98
**Body mass index^d^**				
25.0–29.9 (overweight)	1 [Reference]		1 [Reference]	
30.0–39.9 (obesity)	2.34 (1.75–3.13)	<.001	2.37 (1.77–3.17)	<.001
≥40.0 (extreme obesity)	5.21 (3.22–8.43)	<.001	5.39 (3.32–8.74)	<.001
**Poverty ratio as percentage of federal poverty level^e^**				
≤100	1 [Reference]		1 [Reference]	
100–199	1.22 (0.86–1.72)	.27	1.19 (0.84–1.69)	.32
200–399	1.60 (1.07–2.40)	.02	1.56 (1.04–2.34)	.03
≥400	1.73 (1.06–2.82)	.03	1.64 (0.999–2.7)	.05
**Has health insurance^c^**				
No	—f		1 [Reference]	
Yes	—f	—f	2.20 (1.46–3.32)	<.001
**General health^c^**				
Excellent/very good	1 [Reference]		1 [Reference]	
Good	1.41 (1.03–1.94)	.03	1.45 (1.05–2.00)	.02
Fair/poor	1.94 (1.32–2.85)	.001	2.01 (1.36–2.96)	<.001
**Diabetes diagnosis^c^**				
No	1 [Reference]		1 [Reference]	
Yes	1.82 (1.24–2.67)	.002	1.76 (1.2–2.59)	.004
**Asthma diagnosis^c^**				
No	1 [Reference]		1 [Reference]	
Yes	1.41 (1.03–1.94)	.04	1.36 (0.99–1.88)	.06

Abbreviations: OR, odds ratio; CI, confidence interval.

a Data collected from random-digit–dial telephone surveys in 5 cities in New Jersey in 2009 and 2010: Camden, Newark, New Brunswick, Trenton, and Vineland.

b Multivariate logistic regression used for analysis, controlling for all variables shown in table.

c Self-reported.

d Body mass index calculated as kg/m^2^ according to self-reported height and weight.

e Poverty ratio calculated according to self-reported household income.

f Insurance does not apply in Model 1.

Model 2 showed that respondents who had health insurance had 2.20 higher odds of receiving weight-loss advice than respondents who had no insurance (*P* < .001). We found no major differences in results for income between Model 1 and Model 2. Compared with those who had household incomes at or below 100% of the FPL, those with incomes of 200% to 399% of the FPL had 1.56 higher odds (*P* = .03), and those who had household incomes of 400% of more of the FPL had 1.64 higher odds (*P* = .05) of receiving weight-loss advice from their health care provider. The strength of association of the other covariates did not change between Model 1 and Model 2.

## Discussion

This study examined the association between patient characteristics and reported receipt of weight-loss advice from a health care provider. Several demographic and health-related factors had a significant relationship with receiving weight-loss advice. Our finding that individuals in the lowest income group had significantly lower odds than individuals in higher income groups of receiving weight-loss advice from their health care provider is similar to the findings of previous studies (6,14). Our results for income did not change after adjusting for health insurance, which has been shown to be associated with physicians’ advice in other studies (6,13). Our finding on the relationship between income and weight-loss advice is problematic, because people with the lowest incomes tend to have poorer health outcomes than those with higher incomes (18).

Regardless of health insurance and income, Hispanics were most likely of the 3 racial/ethnic groups to report receiving health care provider advice to lose weight. This finding aligns with the findings in another study, which found that non-white populations were more likely to receive weight-loss advice than white patients (6). However, other studies that examined race did not find an association between race and receiving advice to lose weight (3,8,9,11). A higher prevalence of overweight or obesity among blacks and Hispanics (19) suggests that health care providers may pay more attention to weight problems when counseling black and Hispanic patients. However, we found that only Hispanic respondents (and not non-Hispanic black respondents) had higher odds than non-Hispanic white respondents to report receiving health care provider’s advice to lose weight.

Patients who have risk factors such as obesity, poor health status, diabetes, and asthma would warrant special attention to weight. Our finding that these risk factors increased the odds of receiving weight-loss advice aligns with the findings of other studies. Participants in other studies who had a high BMI were more likely to receive weight-loss advice than participants with a lower BMI (3,8,11). In one study, patients with comorbidities such as diabetes or heart disease had more than 2 times the odds of receiving weight-loss advice (11). Our study found that patients with diabetes had 1.76 higher odds of receiving advice, similar to 1.96 higher odds found in another study (3).

The low rate of reported health care provider advice to lose weight is concerning; only 35% of the sample received advice. However, this rate did increase as BMI increased; 63% of those whose BMI was 40.0 or higher reported receiving weight-loss advice in the previous 12 months, whereas only 22% of overweight (BMI of 25.0–29.9) participants received advice. The rates found in our study are similar to rates found in previous studies: 33% to 65% of the study samples received weight-loss advice (2,3,10,20). However, national guidelines recommend that all obese people receive weight-loss advice, and thus current rates are short of national targets.

Although our study did not examine whether physician characteristics play a role in determining who receives weight-loss advice, one study found that female physicians were more likely than male physicians to provide advice (8). Another study of physician characteristics found that 9% of primary care providers provided 52% of weight-related counseling (21). Health care providers may have many reasons for not providing weight-loss advice. Research on barriers to providing weight-loss advice has been conducted primarily among physicians (22). Many physicians believe they are not effective at providing weight-related counseling and lack the strategies and confidence to provide weight-loss advice (22–24). A lack of time during the medical appointment has also been cited as a barrier to providing weight-loss counseling (22, 25). Many health professionals have negative attitudes toward the overweight or obese population (26, 27). Income biases also exist. Physicians in one study were less likely to rate low-income patients as responsible and intelligent than they were to rate their higher-income counterparts (28). A systematic review found that low-income patients receive less information and less positive feedback from their physician (29). Biases toward patients who are overweight, obese, or low-income, as well as the lack of self-efficacy of physicians in providing effective advice, may contribute to the low prevalence of weight-loss advice.

Our study had several strengths. The inclusion of several health and demographic variables allowed for independent associations of factors not examined in other studies. The study sample included a high proportion of low-income adults from racial/ethnic minority populations — groups not represented in many other similar studies. Our study also had several limitations. One, the data on receiving weight-loss advice were self-reported, and the question asked only about receiving advice in the previous 12 months. Some participants may have received weight-loss advice at an earlier time, and some may not have seen a health care provider in the previous 12 months. Because the answer to this question relied on the respondent’s memory, some participants may have responded incorrectly. However, patient self-report generally has good concordance with medical records (30). Two, because the sample for the study was drawn from communities in New Jersey with a high proportion of low-income, racial/ethnic minority populations, the results are generalizable to similar populations only. Three, the question used to determine receipt of weight-loss advice referred to any health care provider (“a doctor, nurse, or other health professional”), so we do not know if the participant received advice from a physician, a nurse, or other health professional or how much time was spent with that health care provider.

Further work is needed to examine why disparities exist in who receives weight-loss advice. Qualitative studies that interview health care providers may shed light on our findings or the findings of other similar studies. Understanding reasons for our results can inform interventions designed to enhance the knowledge, skills, and self-efficacy of health care providers to offer weight-loss advice to all patients, regardless of income.

## Post-Test Information

To obtain credit, you should first read the journal article. After reading the article, you should be able to answer the following, related, multiple-choice questions. To complete the questions (with a minimum 75% passing score) and earn continuing medical education (CME) credit, please go to http://www.medscape.org/journal/pcd. Credit cannot be obtained for tests completed on paper, although you may use the worksheet below to keep a record of your answers. You must be a registered user on Medscape.org. If you are not registered on Medscape.org, please click on the "Register" link on the right hand side of the website to register. Only one answer is correct for each question. Once you successfully answer all post-test questions you will be able to view and/or print your certificate. For questions regarding the content of this activity, contact the accredited provider, CME@medscape.net. For technical assistance, contact CME@webmd.net. American Medical Association’s Physician’s Recognition Award (AMA PRA) credits are accepted in the US as evidence of participation in CME activities. For further information on this award, please refer to http://www.ama-assn.org/ama/pub/about-ama/awards/ama-physicians-recognition-award.page. The AMA has determined that physicians not licensed in the US who participate in this CME activity are eligible for ***AMA PRA Category 1 Credits™***. Through agreements that the AMA has made with agencies in some countries, AMA PRA credit may be acceptable as evidence of participation in CME activities. If you are not licensed in the US, please complete the questions online, print the AMA PRA CME credit certificate and present it to your national medical association for review.

## Post-Test Questions

### Study Title: Disparities in Who Receives Weight-Loss Advice From a Health Care Provider: Does Income Make a Difference?

#### CME Questions

1. In the current trial, what was the approximate percentage of overweight and obese participants who reported receiving a healthcare provider's advice to lose weight in the past 12 months?
  - 14%
  - 35%
  - 58%
  - 89%
2. Which of the following demographic variables was ***most*** associated with receiving advice to lose weight in the current study?
  - Ages 18 to 34 years
  - Ages 55 years or older
  - Female sex
  - Achievement of a college degree
3. In the current study, which of the following racial and ethnic groups was ***most*** likely to receive advice to lose weight?
  - Hispanics
  - Non-Hispanic blacks
  - Non-Hispanic whites
  - Non-Hispanic Asians
4. In the current study, how did health insurance status and income affect the rate of advice from a healthcare provider regarding weight loss?
  - Having insurance and higher income were both associated with higher rates of weight loss advice
  - Only having insurance was associated with higher rates of weight loss advice
  - Only higher income was associated with higher rates of weight loss advice
  - Having health insurance explained any positive effect of higher income on the rate of weight loss advice

**Table:** Evaluation

**1. The activity supported the learning objectives.**				
**Strongly Disagree**	** **	** **	** **	**Strongly Agree**
1	2	3	4	5
**2. The material was organized clearly for learning to occur.**				
**Strongly Disagree**	** **	** **	** **	**Strongly Agree**
1	2	3	4	5
**3. The content learned from this activity will impact my practice.**				
**Strongly Disagree**	** **	** **	** **	**Strongly Agree**
1	2	3	4	5
**4. The activity was presented objectively and free of commercial bias.**				
**Strongly Disagree**	** **	** **	** **	**Strongly Agree**
1	2	3	4	5

## References

[1] Moyer VA ; US Preventive Services Task Force. Screening for and management of obesity in adults: U.S. Preventive Services Task Force recommendation statement. Ann Intern Med 2012;157(5):373–8. 22733087 10.7326/0003-4819-157-5-201209040-00475

[2] Rodondi N , Humair J-P , Ghali WA , Ruffieux C , Stoianov R , Seematter-Bagnoud L , Counselling overweight and obese patients in primary care: a prospective cohort study. Eur J Cardiovasc Prev Rehabil 2006;13(2):222–8. 10.1097/01.hjr.0000209819.13196.a4 16575276

[3] Breitkopf CR , Egginton JS , Naessens JM , Montori VM , Jatoi A . Who is counseled to lose weight? Survey results and anthropometric data from 3,149 lower socioeconomic women. J Community Health 2012;37(1):202–7. 10.1007/s10900-011-9437-8 21744160 PMC3576430

[4] Lorts C , Ohri-Vachaspati P . Eating behaviors among low-income obese adults in the United States: does health care provider’s advice carry any weight. Prev Med 2016;87:89–94. 10.1016/j.ypmed.2016.02.015 26876632 PMC4884458

[5] Dorsey R , Songer T . Lifestyle behaviors and physician advice for change among overweight and obese adults with prediabetes and diabetes in the United States, 2006. Prev Chronic Dis 2011;8(6):A132. 22005625 PMC3221573

[6] Loureiro ML , Nayga RM Jr . Obesity, weight loss, and physician’s advice. Soc Sci Med 2006;62(10):2458–68. 10.1016/j.socscimed.2005.11.011 16376006

[7] Pool AC , Kraschnewski JL , Cover LA , Lehman EB , Stuckey HL , Hwang KO , The impact of physician weight discussion on weight loss in US adults. Obes Res Clin Pract 2014;8(2):e131–9. 10.1016/j.orcp.2013.03.003 24743008 PMC4264677

[8] Dutton GR , Herman KG , Tan F , Goble M , Dancer-Brown M , Van Vessem N , Patient and physician characteristics associated with the provision of weight loss counseling in primary care. Obes Res Clin Pract 2014;8(2):e123–30. 10.1016/j.orcp.2012.12.004 24743007 PMC3992518

[9] Ko JY , Brown DR , Galuska DA , Zhang J , Blanck HM , Ainsworth BE . Weight loss advice U.S. obese adults receive from health care professionals. Prev Med 2008;47(6):587–92. 10.1016/j.ypmed.2008.09.007 18851991 PMC5176257

[10] Abid A , Galuska D , Khan LK , Gillespie C , Ford ES , Serdula MK . Are healthcare professionals advising obese patients to lose weight? A trend analysis. MedGenMed 2005;7(4):10. 16614632

[11] Bleich SN , Pickett-Blakely O , Cooper LA . Physician practice patterns of obesity diagnosis and weight-related counseling. Patient Educ Couns 2011;82(1):123–9. 10.1016/j.pec.2010.02.018 20303691 PMC2902765

[12] Galuska DA , Will JC , Serdula MK , Ford ES . Are health care professionals advising obese patients to lose weight? JAMA 1999;282(16):1576–8. 10.1001/jama.282.16.1576 10546698

[13] Kraschnewski JL , Sciamanna CN , Stuckey HL , Chuang CH , Lehman EB , Hwang KO , A silent response to the obesity epidemic: decline in US physician weight counseling. Med Care 2013;51(2):186–92. 10.1097/MLR.0b013e3182726c33 23047128

[14] Jackson JE , Doescher MP , Saver BG , Hart LG . Trends in professional advice to lose weight among obese adults, 1994 to 2000. J Gen Intern Med 2005;20(9):814–8. 10.1111/j.1525-1497.2005.0172.x 16117748 PMC1490207

[15] Centers for Disease Control and Prevention. Behavioral Risk Factor Surveillance System 2010 summary data quality report. http://www.cdc.gov/brfss/annual_data/2010/pdf/2010_summary_data_quality_report.pdf. Accessed July 29, 2016.

[16] US National Heart, Lung, and Blood Institute. The practical guide: identification, evaluation, and treatment of overweight and obesity in adults. Bethesda (MD): National Institutes of Health, National Heart, Lung, and Blood Institute, NHLBI Obesity Education Initiative, North American Association for the Study of Obesity; 2000.

[17] McAdams MA , Van Dam RM , Hu FB . Comparison of self-reported and measured BMI as correlates of disease markers in US adults. Obesity (Silver Spring) 2007;15(1):188–96. 10.1038/oby.2007.504 17228047

[18] Braveman PA , Cubbin C , Egerter S , Williams DR , Pamuk E . Socioeconomic disparities in health in the United States: what the patterns tell us. Am J Public Health 2010;100(Suppl 1):S186–96. 10.2105/AJPH.2009.166082 20147693 PMC2837459

[19] Ogden CL , Carroll MD , Fryar CD , Flegal KM . Prevalence of obesity among adults and youth: United States, 2011–2014. NCHS Data Brief 2015;(219):1–8. 26633046

[20] Jackson SE , Wardle J , Johnson F , Finer N , Beeken RJ . The impact of a health professional recommendation on weight loss attempts in overweight and obese British adults: a cross-sectional analysis. BMJ Open 2013;3(11):e003693. 10.1136/bmjopen-2013-003693 24189083 PMC3822310

[21] Kraschnewski JL , Sciamanna CN , Pollak KI , Stuckey HL , Sherwood NE . The epidemiology of weight counseling for adults in the United States: a case of positive deviance. Int J Obes 2013;37(5):751–3. 10.1038/ijo.2012.113 22777541 PMC4429525

[22] Briscoe JS , Berry JA . Barriers to weight loss counseling. J Nurse Pract 2009;5(3):161–7. 10.1016/j.nurpra.2008.08.018

[23] Steeves JA , Liu B , Willis G , Lee R , Smith AW . Physicians’ personal beliefs about weight-related care and their associations with care delivery: the U.S. National Survey of Energy Balance Related Care among Primary Care Physicians. Obes Res Clin Pract 2015;9(3):243–55. 10.1016/j.orcp.2014.08.002 25175671

[24] Kolasa KM , Rickett K . Barriers to providing nutrition counseling cited by physicians: a survey of primary care practitioners. Nutr Clin Pract 2010;25(5):502–9. 10.1177/0884533610380057 20962310

[25] Alexander SC , Østbye T , Pollak KI , Gradison M , Bastian LA , Brouwer RJ . Physicians’ beliefs about discussing obesity: results from focus groups. Am J Health Promot 2007;21(6):498–500. 10.4278/0890-1171-21.6.498 17674636

[26] Puhl RM , Heuer CA . The stigma of obesity: a review and update. Obesity (Silver Spring) 2009;17(5):941–64. 10.1038/oby.2008.636 19165161

[27] Teachman BA , Brownell KD . Implicit anti-fat bias among health professionals: is anyone immune? Int J Obes Relat Metab Disord 2001;25(10):1525–31. 10.1038/sj.ijo.0801745 11673776

[28] van Ryn M , Burke J . The effect of patient race and socio-economic status on physicians’ perceptions of patients. Soc Sci Med 2000;50(6):813–28. 10.1016/S0277-9536(99)00338-X 10695979

[29] Willems S , De Maesschalck S , Deveugele M , Derese A , De Maeseneer J . Socio-economic status of the patient and doctor-patient communication: does it make a difference? Patient Educ Couns 2005;56(2):139–46. 10.1016/j.pec.2004.02.011 15653242

[30] Okura Y , Urban LH , Mahoney DW , Jacobsen SJ , Rodeheffer RJ . Agreement between self-report questionnaires and medical record data was substantial for diabetes, hypertension, myocardial infarction and stroke but not for heart failure. J Clin Epidemiol 2004;57(10):1096–103. 10.1016/j.jclinepi.2004.04.005 15528061

